# Supplementary figures and images for: COVID-19 cases among medical laboratory services staff in South Africa, 2020–2021: A cohort study
Source: PLoS One. 2022 Jun 17;17(6):e0268998. doi: 10.1371/journal.pone.0268998 (PMC9205487; doi:10.1371/journal.pone.0268998)

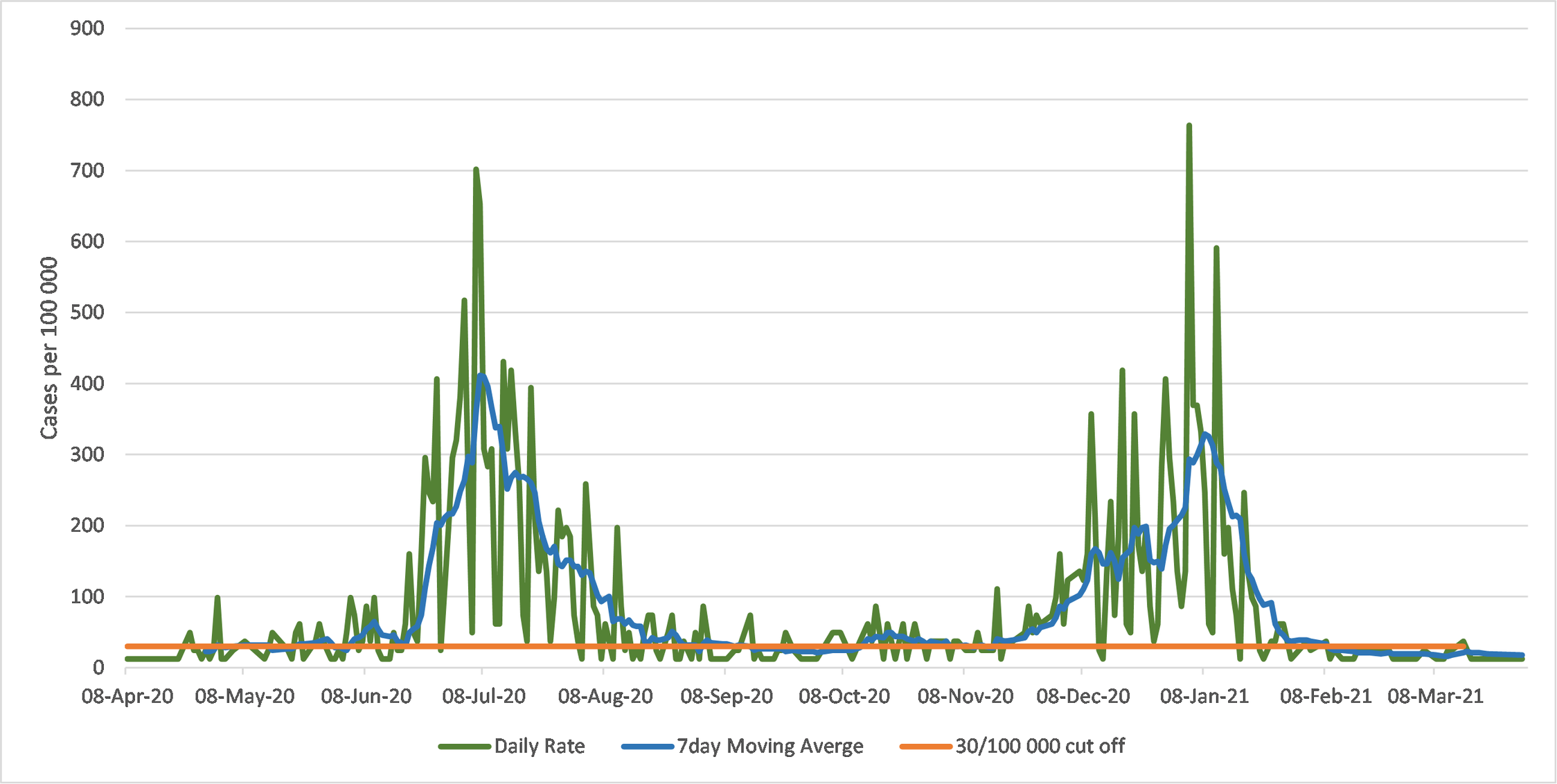

Supplement: S1 Fig — The daily case rate showed a large fluctuation over weekends where testing was seldom available (green) the 7-day moving average showed two clear peaks during the period (blue) the orange line represents the 30/100 000 cut off for a wave. (TIF) [file pone.0268998.s001.tif]
