# Supplementary material for: COVID-19 cases among medical laboratory services staff in South Africa, 2020–2021: A cohort study
Source: PLoS One. 2022 Jun 17;17(6):e0268998. doi: 10.1371/journal.pone.0268998 (PMC9205487; doi:10.1371/journal.pone.0268998)
Supplement: Attachment — Submitted filename: Revision_Letter_PLOSone.docx [file pone.0268998.s002.docx]

Revision Letter

Thank you for allowing us to revise the manuscript and resubmit. We confirm that we have addressed each of the Reviewer’s comments (please see details below). We have also ensured that the manuscript meets the PLOS ONE style requirements. In addition, we have updated the Methods section to include the Data Availability statement. The ethics statement has also been moved to the Methods section, as requested. We have provided the images, as requested.

Journal Requirements

1. Style – The submission style was updated to match the formatting guidelines.
2. Competing interests: One author is involved in establishing the information system used here to provide the data in the local government health departments. This does not alter our adherence to PLOS ONE policies on sharing data and materials. Deidentified participant data containing possibly identifiable data and personal medical data may be obtained on application to the National Health Laboratory Services and with compliance to their requirements and the POPI Act South Africa 2021. The application may be made on registering at <https://aarms.nhls.ac.za/NHLS_AARMS/Public/Default.aspx>
3. Data availability - Deidentified participant data may be obtained from the National Health Laboratory Services on application and compliance with their requirements. https://aarms.nhls.ac.za/NHLS_AARMS/Public/Default.aspx
4. Your ethics statement should only appear in the Methods section of your manuscript. If your ethics statement is written in any section besides the Methods, please delete it from any other section. – ethics statement removed from Acknowledgements and stated in Methods section

**Reviewers Comments**

**Reviewer #1:** COVID-19 cases among medical laboratory services staff in South Africa, 2020-2021: A Cohort Study.

Technical Comments to the Author

The manuscript is written in clear and understandable English and the final message is very strong.

Thank you for this view

Remarks to the Author

Minor comments

1. Remove point from the title. The title should be “COVID-19 cases among medical laboratory services staff in South Africa, 2020-2021: A Cohort Study”

Point removed, title now reads “COVID-19 cases among medical laboratory services staff in South Africa, 2020-2021: A cohort study”

2. Report in the materials and methods section a paragraph on the inclusion and exclusion criteria used in the study.

The paragraph below on population was included in the Methods section, i.e.,

**“Population**

All NHLS staff, including contract workers and experiential students, may report incidents to the surveillance system and, thus, be included in this study. Managers, safety health and environment staff and occupational health nurses may also report incidents reported to them on the surveillance system. Thus, all staff were eligible to be included in this analysis.

**Inclusion criteria**:

- Reported case on OHASIS
- Employed, working or visiting at one of the medical laboratory facilities

**Exclusion criteria**

Age less than 18 years”

3. Move limitations to the Materials and Methods section.

Limitations paragraph moved from the Discussion section to the Methods section

A better description of the study population is needed. Include Supplementary Table 1 as Table 1 in the manuscript and comment on it in the results section.

Table S1 moved to results section and renamed Table 1.

We then included in the results section “The medical laboratory service employs mainly females (68.1%), but no significant relationship was seen between sex and COVID-19 cases in the staff (AOR 0.93 95% CI 0.83 - 1.03). All race groups were represented, where African workers represented 75.5% of the staff. Compared to Caucasian workers, African and Coloured workers had significantly increased odds of COVID-19 infection. The majority of workers were between 30 and 49 years of age (60.8%) and all age groups compared to those less than 29 years showed significantly increased odds of infection. Odds of infection varied significantly between some provinces, with the Eastern Cape reporting the highest odds compared to Gauteng province. (Table 1)”

All following table numbers were also updated.

4. Indicate occupations with risk scores below 0.5 excluded from the analysis.

Although the “COVID-19 Risk Score” that was based on US data excluded jobs with a risk score below 0.5 from further analysis (Table 3), none of the occupations listed in South African OHASIS dataset were excluded from analysis, where occupations with few employees were grouped into similar occupation groups (Table 2). This statement has been added to the main text.

6. The description of the results needs to be improved. For example, figure 2 shows the incidence rates of infection among medical laboratory service personnel by gender, not all data are commented on in the text.

No difference in the incidence rates between men and women was seen. This statement has been added to the main text.

7. The captions of the figures appear within the text. They should be placed at the end of the paragraph in which the figure is cited.

The caption for Fig 1 moved to below the paragraph it is first mentioned in. The remaining two figure captions are correctly placed.

8. Improve the resolution of the figures.

Thank you for noticing. The resolution has been improved to 300dpi

9. Change HCWS to HCWs in conclusion part (line 460). Thank you for catching this typo. It has been corrected to HCWs.

10. HCWs has been critical for monitoring COVID-19 infections and other diseases, not for diagnosis. Please correct (lines 460-461). Diagnosing removed from the sentence.

**Reviewer #2:** Summary: The aim of this study was to show an increased risk of COVID-19 for Medical laboratory workers. Records of COVID-19 laboratory-confirmed positive, in twelve months, were present in System (OHASIS). The results showed that some categories of medical laboratory staff are at increased risk for COVID-19, with the most increased risk were laboratory managers, confirms this is a complex interaction between workplace risk factors. This article is very interesting for the public of this journal. The language of paper is difficult to understand and includes many errors, and the results must be better reported. The main text and the data analysis have to need to improve.

Thank you for your comments. We have completed a spell check to correct spelling errors and have revised grammatical mistakes where needed. The results section has been revised.

1) In Abstract, result section: “… A total of 2091 COVID-19 positive cases (26% of staff) were reported via OHASIS over the twelve months’ period. Already present in materials and methods.

Sentence changed to: A total of 2091 (26% of staff) COVID-19 positive cases were reported.

i.e.,967 (46% of total cases), write in full i.e., write in full ei, such as reported is little clary. ...The number of COVID-19 cases was higher in first wave, i.e.,967 (46% of total cases) than in the second wave 846 (40% of total cases)...this part is very confusional; improve, for example putting the fraction in the parenthesis, and number percentual out of them, n% (n°/n. tot).

Thank you for your suggestion. The sentence was corrected to “The number of COVID-19 cases was higher in the first wave at 46%(967/2091) of cases, than in the second wave 40% (846/2091) of cases”.

2) Line 133: we are missing parentheses and not in Grasset character.

Parentheses included, I am unfortunately not familiar with Grasset characters.

3) Improve the introduction reporting similar studies.

Thank you for this suggestion. Currently this is the only report of COVID-19 incidence in South Africa focusing on the *subcategories* of health workers, such as medical laboratory workers.

4) Line 293: delete r, before the point..

Thank you for your suggestion. The sentence was deleted

5) Line 368: 1 min, no 1m.

The abbreviation was corrected to 1 metre

6) Line 429, 460: correct it .

Line 429 corrected to “These are recommendations that could be implemented within the South African context.”

Line 460 corrected to “HCWs, specifically medical laboratory services staff, have been crucial to monitoring COVID-19 infections and other diseases”

7) Improve the figures, and in my opinion it would be better to remove the totals bar.

Thank you for your comment. The totals bar was removed from Fig 2 and the resolution improved to 300dpi in all figures.
